# Principle vs. Expediency

**Published:** 1892-09

**Authors:** 


### EDITORIAL DEPARTMENT.

### PRINCIPLE VS. EXPEDIENCY.

In the matter of seeking medical legislation our able but weak-kneed contemporary, the *New Orleans Medical and Surgical Journal*, has not only given up the fight against homeopaths, but has surrendered to them; and now proposes to take them into loving embrace as friends and allies, and thus reinforced, to continue the war against all other quacks!

For several years the medical profession of Louisiana with commendable zeal have striven to secure the passage of a law to regulate the practice of medicine, and the *N. O. Medical and Surgical Journal* has backed them, and with them made common cause against all quacks and quackery. They have been defeated each time, however, through the efforts and misrepresentation of the homeopaths, who, as in Texas, when the profession of this State were seeking to secure legislation, cried "persecution," "class legislation," etc., and notwithstanding the last Louisianna bill made many reasonable concessions to the homeopaths, they opposed and defeated it by the same specious pleas. They do not hesitate to say and do say, they will oppose any bill to regulate the practice of medicine emanating from the regular profession.

For our part, regarding them as we do, as outside the profession,—as not being physicians in any sense of the word,—we do not concede to homeopaths the right to be considered at all in the framing of such a law as is needed and sought; and in this the JOURNAL should have the support of all who recognize the code of ethics of our profession as their guide. The code does not recognize any "school" of medicine, and especially forbids consultation with those adhering to any dogma, and calling themselves anything but "physicians."

The *N. O. Medical and Surgical Journal*, however, now comes out and not only in effect recognizes these people as a separate school of medicine,—as physicians, competitors for practice; not equal but superior in sagacity and influence to the medical profession in Louisiana, but acknowledges that they are "too much for them"—nothwithstanding the relative strength numerically of the two parties,—and admits that there "is no use fighting longer for legislation so long as they—the homeopaths—are opposed to it." That journal, therefore, proposes to *join forces* with them and attempt to secure with their *aid* what they have not been able to get with their opposition—a bill to *suppress quackery*.

There is one thing in being defeated; one can, without humiliation, lay down his arms, and even with dignity and self-respect surrender to superior skill or numbers; but it is quite another thing to truckle to the enemy, and lick the hand that smote you.

In this matter the *N. O. Medical and Surgical Journal* has not only given up the fight, but has surrendered the principle upon which it was made. It has done more; by the humiliating confession contained in its leading article on the subject in the August number it has given courage and moral support to the homeopaths which will render them still more presumptuous and persistent in their demands for recognition. The *Southern Journal of Homeopathy*, the slogan of the clans, is jubilant already, and is crowing over the victory in a four column article, bristling with epithets and brimful of bigotry.

It is very desirable to have a law that will prevent the ignorant and unqualified, of whatever class, from attempting to practice medicine, (and according to this JOURNAL'S way of thinking there are more ignorant pretenders sailing under the guise of homeopathic physicians—a misnomer,— a moral impossibility, a contradiction of terms, in fact,—than in all others combined;) but if we cannot have it without the *aid* of those whom it would in a large measure, if properly enforced, exclude; if we cannot have it without surrendering the fundamental principles upon which are based the very idea and object of the law (to suppress quackery), without the aid and co-operation of the largest element of quacks known to the profession, let us cease all efforts in that direction. For what kind of a law "to suppress quackery" would one be which places upon an equal footing with the cultivated physician a lot of more-than-quacks?—men totally uneducated in the principles of rational medicine, and who practice upon a theory long since exploded, which is contrary to reason, common sense and every day experience. Not only have they not studied medicine,—and are therefore totally ignorant of its principles and of rational therapeutics, but they have studied something which inculcates the very opposite of such theory and principles. Yet the majority of them, while calling themselves homeopaths, will prescribe medicines and employ the methods in use in the practice of rational medicine. In this, then, they are more than quacks; they are, knowingly, frauds. If there are any calling themselves homeopaths, and practicing as such, who do not resort to what they call "allopathic" medicines and treatment—thus abandoning the very principle upon which they claim their theory is founded, and attempt its very opposite (of which they are ignorant) let them consider themselves excepted from this catagory; but so far as our knowledge extends they all give up homeopathy when they encounter a serious case, at least one, the tendency of which is to fatal termination, and therefore requires skill and knowledge, and resort to "strong medicine," as they call it.

The other kind of quack, the man who has not studied medicine at all; or, take the one who has studied *at it*, and having obtained a smattering of knowledge, esays to practice; he is at least not a wilful deception. He may be conceited enough to think he knows something about medicine, and may honestly do his best; in other words, he does not know he is an ignoramus; the other fellow does. To seriously propose, therefore, to form an alliance with the horde of homeopathic pretenders is to sacrifice every principle to a mistaken expediency; in the excess of zeal the *N. O. Med. and Surg. Journal* would carry the point at any odds and by any means and at any sacrifice. Why not absorb all other opposition and thus have a clear field? Let the gap down to admit one kind of cattle and you will not be able to bar out any of the others. Let us abandon all idea of "regulating" the practice,—and ask for a law to restrict the practice of medicine to those who can show to the proper authority that they are qualified to exercise the grave prerogative, it matters not when, where nor how that qualification was obtained; make a knowledge of medicine and good moral character the requisite to the privilege. Let us simply ask for a bill to make quackery a penal offense; and if we cannot get it without the assistance of the homeopaths, why, give it up; but for decency's sake, do not compromise the profession by extending to them the long sought recognition as physicians. Else, extend the same recognition to the Christian Scientists and all other like absurdities, and no longer make a pretense of observing the code of ethics.

